# Genes underlying hereditary hearing impairment in humans and in mice

**DOI:** 10.17912/micropub.biology.001728

**Published:** 2025-08-08

**Authors:** Morag A. Lewis, Karen P. Steel

**Affiliations:** 1 Wolfson Sensory, Pain, and Regeneration Centre, King's College London, London, England, United Kingdom

## Abstract

Hearing impairment is a very common disease in the human population, with a high genetic contribution. Here we present a list of genes known to underlie hearing impairment when mutated in humans or in mice. Analysis of the pathways in which the encoded proteins are involved indicates the importance of different signalling pathways to the development and function of the inner ear. The gene list is also useful for identifying candidate genes from human studies such as GWAS or exome sequencing studies.

**Figure 1. Pathways from Reactome which were significantly enriched in hereditary hearing impairment genes:**
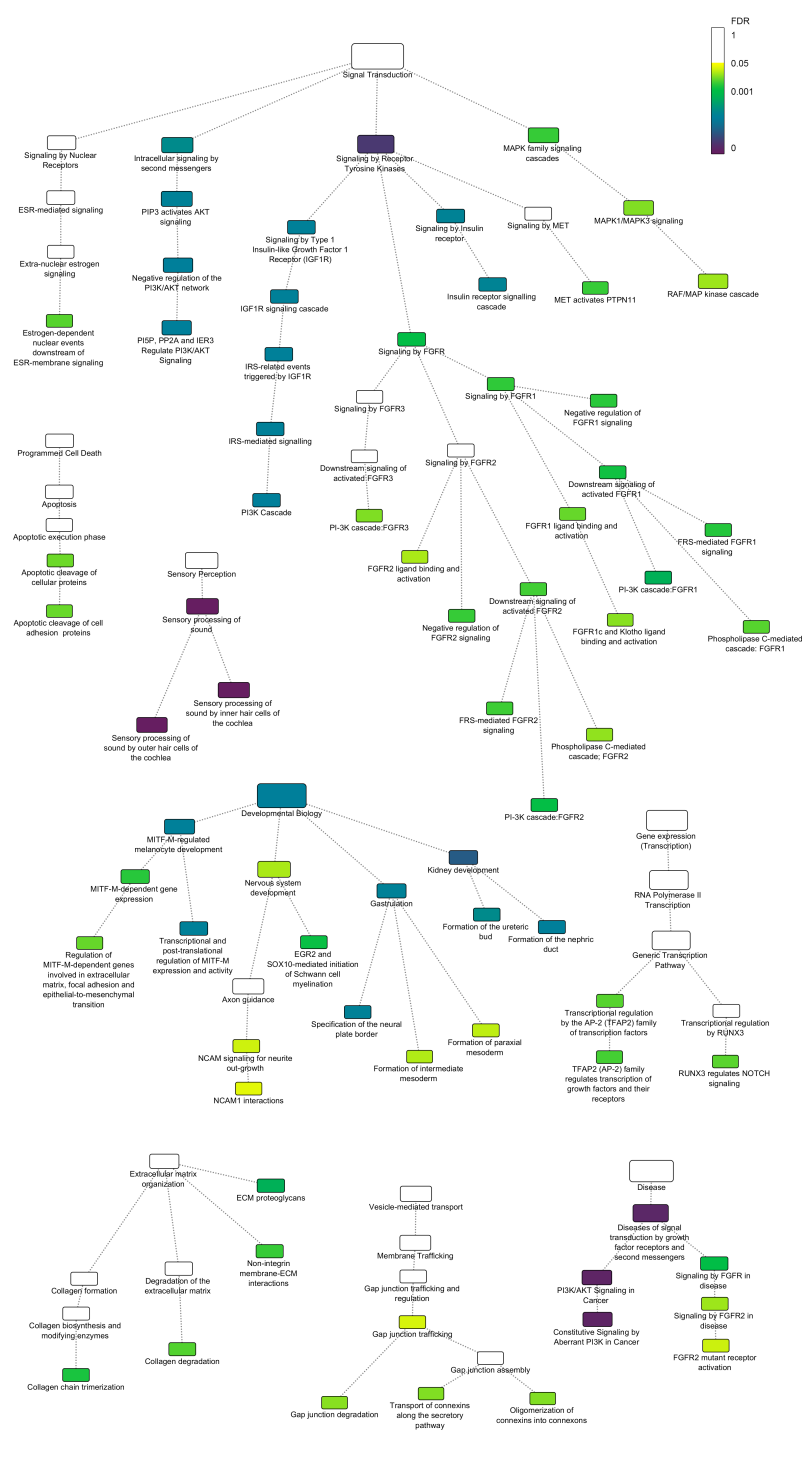
The size of each node represents the number of hereditary hearing impairment genes in that pathway, and the colour indicates the over-representation FDR. Only significantly enriched pathways and their parent pathways are shown, up to the top-level event.

## Description

The inner ear is a complicated, intricate organ, with multiple cell types performing different functions in order to successfully detect sound waves and pass the sensory signal to the brain. Accordingly, hearing impairment may result from any one of many different underlying pathologies, and a treatment for hearing loss, whether small molecules or gene therapy, must take the root cause into account. There is a high contribution of genetics to hearing impairment, with over 150 genes reported to result in hearing loss in humans when mutated (https://hereditaryhearingloss.org/). Knowing which genes are involved should help identify critical pathways for hearing, shed light on potential pathologies, and offer candidate targets for treatment.

In 2019, a large-scale screen of 1,211 mouse genes carrying targeted alleles revealed 38 novel genes involved in deafness (3.14% of the total tested) (Ingham et al. 2019). At the time, 362 human and mouse hearing genes were known, which together with 3.14% of the remaining untested genes suggested there are in total about 1,000 genes involved in hearing. We therefore set out to compile a list of human and mouse hearing genes which, when mutated, result in hearing impairment.

The list was based on published papers, published conference abstracts, and mouse phenotype data made available through the International Mouse Phenotyping Consortium (IMPC; https://www.mousephenotype.org) (Groza et al. 2023). Each gene report was curated before inclusion. The resulting hereditary hearing impairment gene list consists of genes where there is good evidence that mutations result in affected auditory thresholds in humans (117), mice (574), or both humans and mice (136). It should be noted that these are less stringent criteria than those used by the ClinGen Hearing Loss Gene Curation Expert Panel to determine whether or not a gene is linked to hearing impairment in humans (DiStefano et al. 2019).

We analysed the list using the Reactome database (Milacic et al. 2024) and the Reactome pathway analysis tool (Fabregat et al. 2017), and found significant over-representation in many Reactome pathways. Reactome groups pathways into 29 top-level events. Eight of these top-level events included reactions with a significant over-representation of hereditary hearing impairment genes, for example Sensory perception, Developmental Biology, and Signal Transduction (Figure 1). By far the most populated top-level event was Signal Transduction, pointing to the importance of different signalling pathways in both the development and function of the inner ear. Signal transduction sub-pathways included ESR-mediated signalling, FGFR signalling, PI3K/AKT signalling, and MAPK family signalling. It should be noted that these results do not include all pathways implicated in hearing. For example, the S1P pathway, which involves multiple components known to be essential for hearing (Chen et al. 2014; Chen et al. 2024; Ingham et al. 2016), is not currently represented in Reactome. Indeed, while only eight top-level events had subpathways in which hereditary hearing impairment genes were significantly over-represented, there was only one (Digestion and absorption) in which none of the hereditary hearing impairment genes were present at all, indicating the wide range of genes and molecular pathways required for development, maintenance and function of the ear.

Other uses for the list include identifying good gene candidates. For example, DFNA24, which causes mild-to-profound hearing loss affecting mainly the high frequencies, was found to localise to chr4:182,300,001-190,214,555 in a large human pedigree (Hafner et al. 2000). There are two genes from the list in that region, both orthologues of mouse deafness genes: *CASP3* (Takahashi et al. 2001) and *DCTD* (https://www.mousephenotype.org/data/genes/MGI:2444529). These would be good candidates to investigate. The list is also valuable for prioritising candidate genes identified in human studies. In GWAS, for example, a significant locus may not map directly to a specific gene, often yielding a set of nearby genes to consider. Similarly, exome sequencing frequently produces a list of candidate genes with variants. The hereditary hearing impairment gene list can also help assess whether a given list of candidate genes is biologically relevant for hearing (Lewis et al. 2022).

It is possible that our hereditary hearing impairment gene list could shed light on other conditions which are frequently comorbid with hearing impairment, such as kidney disease and retinal conditions. We obtained lists of relevant genes from ClinGen (https://www.clinicalgenome.org) to test this possibility. Four of the hereditary hearing impairment genes were found in the kidney gene list ( *PKD1* , *JAG1* , *DCDC2* , *CYS1* ), and eight hereditary hearing impairment genes were found in the retinal gene list ( *KCNJ13* , *MKKS* , *BBS4* , *LRP5* , *TTC8* , *BBS1* , *FZD4* , *TOPORS* ). Neither of these overlaps was significant (assessed using hypergeometric tests). This is probably because the criteria for inclusion in the kidney and retina lists are much more stringent, and are restricted to human disease associations, so the lists are much smaller (112 retinal disease genes and 64 kidney cystic and ciliopathy genes, compared to 853 human hereditary hearing impairment genes (including human orthologues of mouse hearing impairment genes)). Our hereditary hearing impairment gene list may be useful for identifying candidates for conditions frequently comorbid with hearing impairment, but follow-up studies will always be essential.

The list is available at Zenodo ( https://doi.org/10.5281/zenodo.15705587 ) and will be updated periodically.

## Methods

The gene list is based on publications, including published conference abstracts and online datasets, identified through our own research and the following online resources. The Hereditary Hearing Loss Homepage describes genes implicated in syndromic and non-syndromic human hearing impairment (https://hereditaryhearingloss.org/). OMIM (https://omim.org/) and the Mouse Genome Informatics database (https://www.informatics.jax.org/) allow searching by phenotype. For each gene so identified, the original publication was checked. In some cases genes were not included if hearing impairment was not a consistent feature associated with mutant alleles (eg *MANB* , identified through OMIM, where hearing impairment was reported once in 9 example case studies). We also made use of the International Knockout Mouse Consortium data, made available via the IMPC (https://www.mousephenotype.org; (Groza et al. 2023)). The auditory brainstem response test was carried out on a subset of mutant mice, but the automated calling is not always accurate. For example, mice carrying the *Ncoa2 ^em1(IMPC)Mbp^* mutant allele display thresholds not appreciably different from the control animals, but are listed as having abnormal auditory brainstem responses (https://www.mousephenotype.org/data/genes/MGI:1276533), while mice homozygous for the *Myo7a ^tm1a(EUCOMM)Wtsi^* allele are completely deaf, but are not identified as having a hearing phenotype (https://www.mousephenotype.org/data/genes/MGI:104510). Thus, all genes reported by the IMPC to result in altered hearing thresholds were checked for differences over 10dB, with small standard deviations, prior to inclusion. Human/mouse orthologues were identified using Ensembl (https://www.ensembl.org/; (Dyer et al. 2025)). Gene list analysis was carried out using Reactome (Fabregat et al. 2017; Milacic et al. 2024), with the human gene IDs and no interactors selected. The full Reactome results are available as Extended Data.

## Data Availability

Description: Reactome pathway analysis over-representation results. Resource Type: Dataset. DOI: https://doi.org/10.22002/3sj1b-0zb53
